# Comparison of *Mycobacterium ulcerans* (Buruli ulcer) and *Leptospira sp*. (Leptospirosis) dynamics in urban and rural settings

**DOI:** 10.1371/journal.pntd.0007074

**Published:** 2019-01-07

**Authors:** Marine Combe, Rodolphe Elie Gozlan, Soushieta Jagadesh, Camilla Jensen Velvin, Rolland Ruffine, Magalie Pierre Demar, Pierre Couppié, Felix Djossou, Mathieu Nacher, Loïc Epelboin

**Affiliations:** 1 ISEM UMR226, Université de Montpellier, CNRS, IRD, EPHE, Montpellier, France; 2 MIVEGEC, IRD, CNRS, Université de Montpellier, Montpellier, Centre IRD de Cayenne, Guyane française; 3 Equipe EA 3593, Ecosystèmes amazoniens et pathologie tropicale, Université de la Guyane, Cayenne, Guyane française; 4 Laboratoire hospitalo-universitaire de parasitologie mycologie, Centre hospitalier Andrée Rosemon, Guyane française; 5 Service de Dermatologie, Cayenne Hospital, rue des Flamboyant, Cayenne, French Guiana, France; 6 Unité des maladies infectieuses et tropicales, Centre hospitalier Andrée Rosemon, av des Flamboyants, Guyane française; 7 Centre d’investigation clinique (CIC Inserm 1424), Centre hospitalier Andrée Rosemon, Guyane française; Institute for Disease Modeling, UNITED STATES

## Abstract

**Background:**

Zoonotic pathogens respond to changes in host range and/or pathogen, vector and host ecology. Environmental changes (biodiversity, habitat changes, variability in climate), even at a local level, lead to variability in environmental pathogen dynamics and can facilitate their transmission from natural reservoirs to new susceptible hosts. Whilst the environmental dynamics of aquatic bacteria are directly linked to seasonal changes of their habitat they also rely on the ecological processes underpining their transmission. However data allowing the comparison of these ecological processes are lacking. Here we compared the environmental dynamics of generalist and vector-borne aquatic bacterial pathogens in the same unit of time and space, and across rural and urban habitats in French Guiana (South America).

**Principal findings:**

Using *Leptospira sp*. and *Mycobacterium ulcerans* we performed an environmental survey that allowed the detection of both pathogens in urban *vs*. rural areas, and during rainy *vs*. dry weather conditions. All samples were subjected to qPCR amplifications of LipL32 (*Leptospira sp*.) and *IS*2404 and KR (*M*. *ulcerans*) genetic markers. We found *(i)* a greater presence of *M*. *ulcerans* in rural areas compared with *Leptospira sp*., *(ii)* that modified urban environments were more favourable to the establishment of both pathogens, *(iii)* that *Leptospira sp*. presence was enhanced during the rainy season and *M*. *ulcerans* during the dry period, and *(iv)* differences in the spatial distribution of both bacteria across urban sites, probably due to the mode of dissemination of each pathogen in the environment.

**Conclusions:**

We propose that in French Guiana simplified and modified urban ecosystems might favour leptospirosis and Buruli ulcer emergence and transmission. Moreover, disease risk was also constrained by seasonality. We suggest that the prevention of aquatic bacterial disease emergence in impoverished urban areas of developing countries would benefit from seasonal diseases targeted surveys, which would maximise limited budgets from cash-strapped health agencies.

## Author summary

Many emerging pathogens are zoonotic and transmit from their abiotic reservoir to wild animals, domesticated animals and humans. It is now well known that environmental changes lead to variability in their dynamics in the environment and contribute to changes in the infectious risk. Many aquatic bacteria are responsable for major public health concerns, and more importantly in developing countries where access to drinking-water and sanitation is often limited. Whilst their environmental dynamics are directly linked to seasonal changes of their habitat, they also rely on the ecological processes underpining their transmission, i.e. directly transmitted *vs*. vector-borne. However, few studies have compared such environmental dynamics despite the fact that it would help to better characterise the infectious risk in the environment, as well as to better monitor the emergence of infectious diseases. Our aim was to provide data on the prevalence of generalist *vs*. vector-borne aquatic bacterial pathogens in the environment that would further allow the comparison of their environmental dynamics in the same unit of time and space, and across rural and urban habitats. We showed that urbanization and seasonality are two important factors underlying Buruli ulcer and leptospirosis disease emergence in French Guiana (South America), and propose that the mode of transmission of such environmental pathogens might have a detrimental role in disseminating the infectious agent in the environment.

## Introduction

During the last decades infectious diseases have considerably increased in incidence and new pathogens have emerged and/or re-emerged [1,2]. The majority of known pathogenic species are represented by human pathogens (61%), and most of these are zoonotic [3]. Zoonotic pathogens are widespread in the environment and often transmit from their abiotic reservoir to wild animals (biotic reservoir) but also to domesticated animals and humans (susceptible hosts). Moreover, emerging/re-emerging pathogens are opportunists and respond to changes in host range and/or pathogen, vector and host ecology [3,4]. Thus environmental changes, even at a local level, leads to variability in pathogen dynamics in the environment and contributes to changes in the infectious risk [5].

Biodiversity changes through fragmentation and degradation of natural habitats, and particularly in tropical areas, increase contacts between wildlife, domestic animals and humans, facilitating the transmission of environmental pathogens from natural reservoirs to new susceptible hosts [2]. Biodiversity loss is now well recognized to be associated with the increase in emergence of infectious diseases [2,6,7]. Urbanization and agricultural intensification change land-use, population size and population density, but also impact the interactions between pathogens-vectors-hosts and thus may affect the spread of environmental pathogens [7,8]. Also, whilst the exact transmission routes of many tropical diseases remain unclear, variability in climate, even at a local scale, has been reported to affect the prevalence of infectious pathogens in the environment, as well as their transmission dynamics [9,10].

Many aquatic bacteria are responsable for major public health concerns, and more importantly in developing countries where access to drinking-water and sanitation is often limited (for example *Vibrio cholerae*, *Salmonella enterica*, *Shigella sp*., *Leptospira sp*., *Mycobacterium sp*., etc.) [11]. Whilst the environmental dynamics of such pathogens are directly linked to their habitat seasonal changes (i.e. water temperature, *pH*, oxygen level, salinity, sedimentation/turbidity, presence of biofilm, rainfall patterns, etc.), they also rely on the ecological processes underpining their transmission. For instance, a bacterium directly transmitted from the environment might be more constrained by the local habitat parameters when compared to a bacterium disseminated through a vector, thus depending on the availability, abundance and ecology of that particular vector. However, few studies have described such environmental dynamics in a singular unit of time and space, despite the fact that such description would help to better characterize the infectious risk in the environment (e.g. urban *vs*. contryside), as well as to better monitor the emergence of infectious diseases.

*Mycobacterium ulcerans* and *Leptospira sp*. are two pathogenic bacteria found in tropical areas that are accidentally transmitted to humans from the aquatic environment [5,8,12]. These pathogens are responsible for Buruli ulcer and leptospirosis, respectively, that account for significant morbidity and mortalities among impoverished urban settlements [5,8,12,13]. Whilst *M*. *ulcerans* is considered as a generalist pathogen (i.e. associated with different taxa of the aquatic trophic network) with no clear transmission routes to humans [8], *Leptospira sp*. are transmitted to humans through contact of skin lesions or mucous membranes with contaminated surface water or soil [12], but is mainly disseminated in the environment *via* urinary secretions of rodent populations which act as a major reservoir for pathogenic leptospires [5]. Land-use changes (i.e. deforestation) in tropical areas were correlated with increased prevalences of *M*. *ulcerans* in the environment [14]. Urbanization, associated with increased population density and inadequate sanitation (precarious sewer systems and trash accumulation), favours rodent populations expansion and thus increases leptospirosis risk [5]. Also, local weather patterns are important drivers for both diseases transmission since Buruli ulcer cases are associated to rainfall patterns, with cases occurring during the dry season following a flooding event [9], and leptospirosis outbreaks frequently occur during periods of seasonal rainfall and flooding [15,16,17]. Therefore, Amazonian environmental conditions are highly favorable for the persistance of both bacteria in aquatic systems [18,19] and disease cases are notably reported in French Guiana (South America) [13,20]. Focusing on these two aquatic pathogens as model systems we performed an environmental survey along the French Guiana coastline with the objective to test the following hypothesis:

- Prevalences and distributions of both bacteria in the environment differ in urbanized and natural habitats, with urbanization either increasing or reducing disease risk. Here we described the potential impact of urbanization on the risk of leptospirosis and Buruli ulcer.
- Seasonality (drought and rain) has a strong effect on the environmental dynamics of both bacteria. Here we tested if local variability in weather patterns could constitute a major determinant in disease risk.

Finally, we discussed about the likely impact of the transmission mode, e.g. vector-borne *versus* generalist pathogen, on the spatio-temporal dynamics of both bacteria in the environment.

## Materials and methods

### Environmental survey

**Fig 1:**
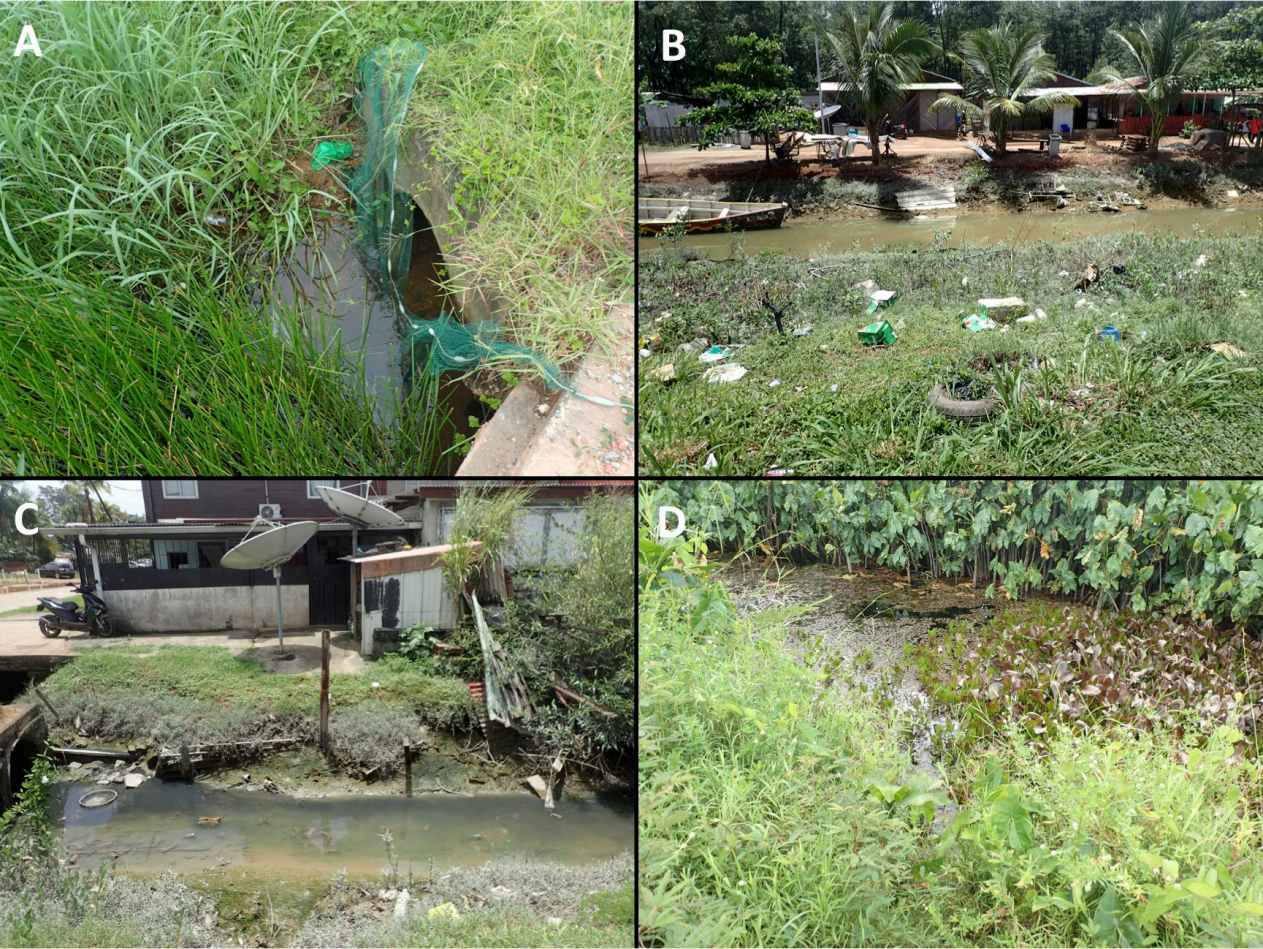
Diversity of environmental sites found positive for *Leptospira sp*. and *M*. *ulcerans* DNA during the period surveyed in French Guiana. A: urban site U30; B: urban site U1; C: urban site U4; D: rural site R9.

Between november 2015 and march 2017 a total of 18 rural aquatic sites were sampled monthly for water and sediments. Rural sites were located along the French Guiana coastline and also along the Sinnamary river. These sites were selected based on previous sampling campaigns in French Guiana [19] and represented ponds and oxbows characterized by low water level and stagnant water, either shaded or sunny, composed of a community of aquatic taxa and surrounded either by vegetation or a dense tropical rainforest (Fig 1, Table 1).

**Table 1:** Site characteristics. Each site was tested for the presence of *Leptospira sp*. DNA and *M*. *ulcerans* DNA. Urban samples were collected from september 2016 to october 2017, while rural samples were collected over the period november 2015-march 2017. When a site was found positive for the bacteria’s DNA at least one time we indicated it as positive +. When a site was always found negative we added the symbol - See Supporting Information S1 Table for qPCR CT-values.

Site	Area	Latitude	Longitude	Sample type	*Leptospira sp*.	*M*. *ulcerans*
U1	Urban	4.93037	-52.33123	Water/Sediment	+	+
U2	Urban	4.93052	-52.33151	Water/Sediment	-	-
U3	Urban	4.93084	-52.33136	Water/Sediment	-	-
U4	Urban	4.92697	-52.33201	Water/Sediment	+	+
U5	Urban	4.92958	-52.33241	Water/Sediment	+	+
U6	Urban	4.92951	-52.33275	Water/Sediment	-	+
U7	Urban	4.93505	-52.32441	Sediment	-	-
U8	Urban	4.93507	-52.31910	Sediment	+	+
U9	Urban	4.93318	-52.31406	Sediment	+	-
U10	Urban	4.93754	-52.29153	Sediment	+	-
U11	Urban	4.93876	-52.29340	Sediment	-	-
U12	Urban	4.93359	-52.28977	Sediment	-	-
U13	Urban	4.93902	-52.29546	Sediment	-	-
U14	Urban	4.93756	-52.31689	Sediment	-	-
U15	Urban	4.92430	-52.31720	Sediment	-	-
U16	Urban	4.91917	-52.31267	Sediment	+	-
U17	Urban	4.92512	-52.31252	Sediment	+	-
U18	Urban	4.91295	-52.27134	Sediment	-	-
U19	Urban	4.91655	-52.27012	Sediment	-	-
U20	Urban	4.88598	-52.26323	Sediment	+	-
U21	Urban	4.86061	-52.25690	Sediment	-	-
U22	Urban	4.88733	-52.27473	Sediment	-	-
U23	Urban	4.88917	-52.27728	Sediment	-	-
U24	Urban	4.89426	-52.28606	Sediment	+	-
U25	Urban	4.89277	-52.28524	Sediment	+	-
U26	Urban	4.90125	-52.27906	Sediment	-	-
U27	Urban	4.89999	-52.27838	Sediment	-	-
U28	Urban	4.90269	-52.27752	Sediment	-	-
U29	Urban	4.90765	-52.28439	Sediment	-	-
U30	Urban	4.91411	-52.28640	Sediment	+	+
U31	Urban	4.93293	-52.33434	Sediment	-	+
U32	Urban	4.93108	-52.33344	Sediment	-	+
U33	Urban	4.92908	-52.33273	Sediment	+	+
U34	Urban	4.93546	-52.33200	Sediment	+	-
U35	Urban	4.93514	-52.33314	Sediment	+	+
U36	Urban	4.93687	-52.32674	Sediment	+	+
U37	Urban	4.93896	-52.29173	Sediment	-	-
U38	Urban	4.93357	-52.33455	Sediment	-	-
U39	Urban	4.93278	-52.33372	Sediment	-	-
U40	Urban	4.93132	-52.29587	Sediment	+	-
U41	Urban	4.93198	-52.29520	Sediment	-	-
U42	Urban	4.89001	-52.27864	Sediment	-	-
U43	Urban	4.90012	-52.26398	Sediment	+	-
U44	Urban	4.89958	-52.26347	Sediment	+	-
U45	Urban	4.86795	-52.27754	Sediment	+	-
U46	Urban	4.86671	-52.28035	Sediment	-	-
U47	Urban	4.87460	-52.33138	Sediment	+	-
U48	Urban	4.87628	-52.33044	Sediment	+	-
U49	Urban	4.84565	-52.32909	Sediment	+	-
U50	Urban	4.84776	-52.32204	Sediment	+	-
U51	Urban	4.88733	-52.27473	Sediment	-	-
R1	Rural	4.83808	-52.35325	Water/Sediment	-	-
R2	Rural	4.62168	-52.93251	Water/Sediment	-	-
R3	Rural	4.61602	-52.90554	Water/Sediment	-	-
R4	Rural	4.55858	-52.90674	Water/Sediment	-	-
R5	Rural	4.54898	-52.89271	Water/Sediment	-	+
R6	Rural	4.54141	-52.88958	Water/Sediment	-	-
R7	Rural	4.50403	-52.87810	Water/Sediment	-	-
R8	Rural	5.16008	-52.89310	Water/Sediment	-	-
R9	Rural	5.39410	-52.99201	Water/Sediment	+	+
R10	Rural	5.31043	-53.04884	Water/Sediment	-	-
R11	Rural	5.31534	-53.04665	Water/Sediment	-	-
R12	Rural	5.31910	-53.04499	Water/Sediment	-	-
R13	Rural	5.32946	-53.03592	Water/Sediment	-	+
R14	Rural	5.44505	-53.15818	Water/Sediment	-	-
R15	Rural	5.07358	-53.05289	Water/Sediment	-	-
R16	Rural	5.34028	-52.92822	Sediment	-	-
R17	Rural	5.03535	-52.51648	Sediment	-	+
R18	Rural	4.86015	-52.27552	Sediment	-	-

In parallel, 51 urban water bodies were also sampled for water and/or sediments from september 2016 to october 2017 among three urban centers: Cayenne (2441.27 inhabitants/km^2^), Rémire-Montjoly (519.97 inhabitants/km^2^) and Matoury (236.37 inhabitants/km^2^) (Fig 1, Table 1). Urban sites were selected based on the location of leptospirosis and Buruli ulcer cases and corresponded to small ditches. Disease cases were mapped and we selected water bodies that fitted the following conditions: (i) being close to Buruli ulcer and/or leptospirosis cases and (ii) showing the ecological conditions prone to sustain the bacteria in the environment (i.e. small water body with low water level and biofilm development for *M*. *ulcerans*, and prone to harbour rodents for *Leptospira sp*.). Water was collected in the middle of the water body, from the water column between 0–1 m below the surface and kept in 1.5 L plastic bottles stored on ice and transported to the laboratory. Also the first layer of sediments (0–1 cm depth) was collected in 30 mL tubes stored on ice and transported to the laboratory. Samples were kept at 4°C until DNA extractions (performed within 24 h for water and 48 h for sediments). Moreover all sites were surveyed during both the dry (september 2016/october 2016/july 2017/october 2017) and the rainy (february and may 2017) seasons in order to compare the distributions and prevalences of both environmental pathogens in space and time (Table 2).

**Table 2:** Number of urban sites found positive for *Leptospira sp*. and *M*. *ulcerans* DNA during the 6 sampling periods. The number of positive sites over the total number of sites tested, the prevalence and the climatic conditions are indicated. Months where no samples were collected are indicated by a dash. Climatic data were recorded at the Cayenne climatic station (Météo France) (http://www.meteofrance.com/climat/outremer/cayenne).

		*Leptospira sp*.	*M*. *ulcerans*		
Month	Season	Positive (prevalence)	Positive (prevalence)	Rainfall (mm)	Mean temperature (°C)
September 2016	Dry	1/6 (16.7%)	4/6 (66.7%)	35.4	27.9
October 2016	Dry	2/34 (5.9%)	4/34 (11.8%)	0.2	28.5
November 2016	Dry	-	-	34.8	28.5
December 2016	Rainy	-	-	474.0	27.3
January 2017	Rainy	-	-	286.7	26.9
February 2017	Rainy	17/36 (47.2%)	4/36 (11.1%)	270.4	26.7
March 2017	Rainy	-	-	300.8	27.1
April 2017	Rainy	-	-	315.4	27.8
May 2017	Rainy	8/36 (22.2%)	2/36 (5.6%)	666.7	27.0
June 2017	Rainy	-	-	267.7	27.2
July 2017	Dry	2/36 (5.6%)	5/36 (13.9%)	97.4	27.2
August 2017	Dry	-	-	4.0	28.2
September 2017	Dry	-	-	36.6	28.4
October 2017	Dry	4/21 (19.0%)	8/21 (38.1%)	41.5	28.4

### Human cases

Disease cases were reported at the Cayenne Hospital and were provided by Dr. Loïc Epelboin (Infectious Disease Unit) and Prof. Pierre Couppié (Dermatology Unit). Leptospirosis database provided by Dr. Loïc Epelboin was based on the diagnosis for each patient that has been reported at the Cayenne Hospital and the Centre National de Référence de la Leptospirose at the Pasteur Institute in Paris. Leptospirosis cases reported in French Guiana were analyzed and made available for the period 2014–2017 by the Cayenne Hospital, the Pasteur Institute in Cayenne and the Biomnis laboratory. Buruli ulcer database was built by Prof. Pierre Couppié and colleagues and cases were available from 1969 to 2017. However to be consistent in our comparison of disease cases and positive environmental sites we mapped only cases diagnosed between 2015–2017, occurring thus over the same timescale.

### DNA extractions

Water samples (1.5 L) were first filtered onto 1.6 μm GF/C glass microfiber filters (Whatman) and then through 0.45 μm cellulose nitrate membrane filters (Merck Millipore). These later filters were air dried and kept at -20°C until further analysis. Total DNA was extracted from filtered water using the DNeasy PowerWater extraction kit (Qiagen) following the manufacturer’s recommendations. For sediment samples, 250 mg of sediments were used to extract DNA using the DNeasy PowerSoil extraction kit (Qiagen). Extracted DNA was kept at -20°C.

### Real-time quantitative PCR

To detect and quantify *M*. *ulcerans* DNA in environmental samples, we performed two TaqMan qPCR runs; one targetting the insertion sequence *IS*2404 and one targetting the ketoreductase B (KR) domain of the mycolactone polyketide synthase gene that is specifically found in the virulence plasmid of *M*. *ulcerans* strains. To amplify *IS*2404 genetic marker, we used the following primer and probes: *IS*2404 forward primer 5’-ATTGGTGCCGATCGAGTTG-3’, *IS*2404 reverse primer 5’-TCGCTTTGGCGCGTAAA-3’ and *IS*2404 probe FAM-CACCACGCAGCATTCTTGCCGT-BHQ1 [21]. For KR amplification we used KR forward primer 5’-TCACGGCCTGCGATATCA-3’, KR reverse primer 5’-TTGTGTGGGCACTGAATTGAC-3’, and KR probe FAM-ACCCCGAAGCACTG-MGBNFQ [19]. The qPCR reaction consisted of 1X TaqMan Gene Expression Master Mix (LifeTechnologies), 0.3 μM (final concentration) of each primer, 0.1 μM (final concentration) of the probe, 5 μl of DNA and water adjusted to a final volume per reaction of 25 μl. For KR we followed the same protocol except that we used the probe at a final concentration of 0.25 μM. An internal positive control (IPC) was added in each *IS*2404 reaction in order to test for the presence of PCR inhibitors in the environmental samples. In each qPCR plate, a positive (*M*. *ulcerans* DNA at a concentration of 10^5^ bacteria/mL) and negative (DNA replaced by water) controls were included. The positive control for *M*. *ulcerans* consisted of genomic DNA purified from a cultured strain from French Guiana (strain 1G897) and provided by Laurent Marsollier (ATOMYCA, Université d’Angers). This positive control was also used in our study to run standard curves based on serial dilutions of purified DNA from 10^5^ to 10^0^ bacteria/mL (in triplicates). Standard curves allowed us to determine a threshold value above which we considered our samples as negative (CT-values > 38). The assays were run in duplicates on an Applied Biosystems 7300 Real Time PCR system, with the following program: one cycle at 50°C for 2 min, one cycle at 95°C for 10 min, followed by 45 cycles at 95°C for 15 sec and at 60°C for 1 min. Only samples with cycle threshold values < 38 for both *IS*2404 and KR markers in 1 out of 2 replicates were considered as positives. In all assays the negative controls remained negative.

To date, 22 species of *Leptospira* have been described and arranged into 3 groups based on their pathogenicity; pathogenic species (*L*. *interrogans*, *L*. *kirschneri*, *L*. *borgpetersenii*, *L*. *mayottensis*, *L*. *santarosai*, *L*. *noguchii*, *L*. *weilii*, *L*. *alexanderi*, *L*. *kmetyi*, *L*. *alstonii*), intermediate species of unclear or low pathogenicity (*L*. *broomii*, *L*. *fainei*, *L*. *inadai*, *L*. *licerasiae*, *L*. *wolffii*), and saprophytic species which are free-living cells in water and soil and are not infectious (*L*. *biflexa*, *L*. *idonii*, *L*. *meyeri*, *L*. *terpstrae*, *L*. *vanthielli*, *L*. *wolbachii*, *L*. *yanagawae*) [22]. Whilst pathogenic and intermediate *Leptospira* species are infectious for humans or animals [22], most diagnotic PCR tools only detect *Leptospira* from the pathogenic cluster and fail to detect intermediate species [23]. Among these tools the TaqMan qPCR assay targetting the *lipL32* gene is commonly used to detect pathogenic *Leptospira* [5,23,24] since it encodes outer membrane proteins and virulence factors found in pathogenic species [22]. This qPCR assay has been optimized for both sensitivity and specificity, allowing thus to detect and characterize *Leptospira sp*. in low number or in samples that contain high concentrations of non-*Leptospira* DNA [22,23]. However since this target gene is highly conserved among *Leptospira* species it does not allow the discrimination between species. Therefore, to detect the presence of *Leptospira sp*. DNA we performed a qPCR targetting the *lipL32* gene. To do so, we used forward primer LipL32-45F 5’-AAGCATTACCGCTTGTGGTG-3’, reverse primer LipL32-Rb 5’-GAACTCCCATTTCAGCGAT-3’ and the probe LipL32-189P FAM-AAAGCCAGGACAAGCGCCG-BHQ1 [23]. The qPCR reaction consisted of 1X TaqMan Gene Expression Master Mix (LifeTechnologies), 0.7 μM (final concentration) of each primer, 0.15 μM (final concentration) of the probe, 5 μl of DNA and water adjusted to a final volume per reaction of 25 μl. In each qPCR plate, a positive (*L*. *santarosai* DNA at a concentration of 10^2^ bacteria/mL) and negative (DNA replaced by water) controls were included. The positive control for *Leptospira sp*. consisted of genomic DNA of the strain *L*. *santarosai* that was provided by the Pasteur Institute in Paris (Pascale Bourhy, Centre National de Référence de la Leptospirose). Standard curves were run with serial dilutions of genomic DNA from *L*. *santarosai* from 10^5^ to 10^0^ bacteria/mL (in triplicates). The assays were run on an Applied Biosystems 7300 Real Time PCR system, with the following program: one cycle at 50°C for 2 min, one cycle at 95°C for 10 min, followed by 45 cycles at 95°C for 15 sec and at 60°C for 1 min. Only samples with cycle threshold values < 40 were considered as positives. In all assays the negative controls remained negative.

### Bacterial prevalences

The prevalences of *Leptospira sp*. and *M*. *ulcerans* in the environment were calculated for each sampling period based on the ratio between the number of sites found positive and the total number of sites tested (%). Whilst we measured the quantity of DNA rather than the quantity of live bacteria in the environment [25], the variation in DNA concentration between sites and between each sampling periods was used as a proxy of bacterial abundances in the environment.

### Statistics & maps

Statistics were performed with R version 3.5.1 (R Development Core Team). We used raw data to perform a logistic binomial regression (package stats, function glm) in order to test for the effect of seasonality (dry and rainy seasons) on the pathogen’s presence in the environment (significance threshold: p-value < 0.05). Maps were created with QGIS (Las Palmas, version 2.18.20).

## Results

### Impact of urbanization on disease risk

**Fig 2:**
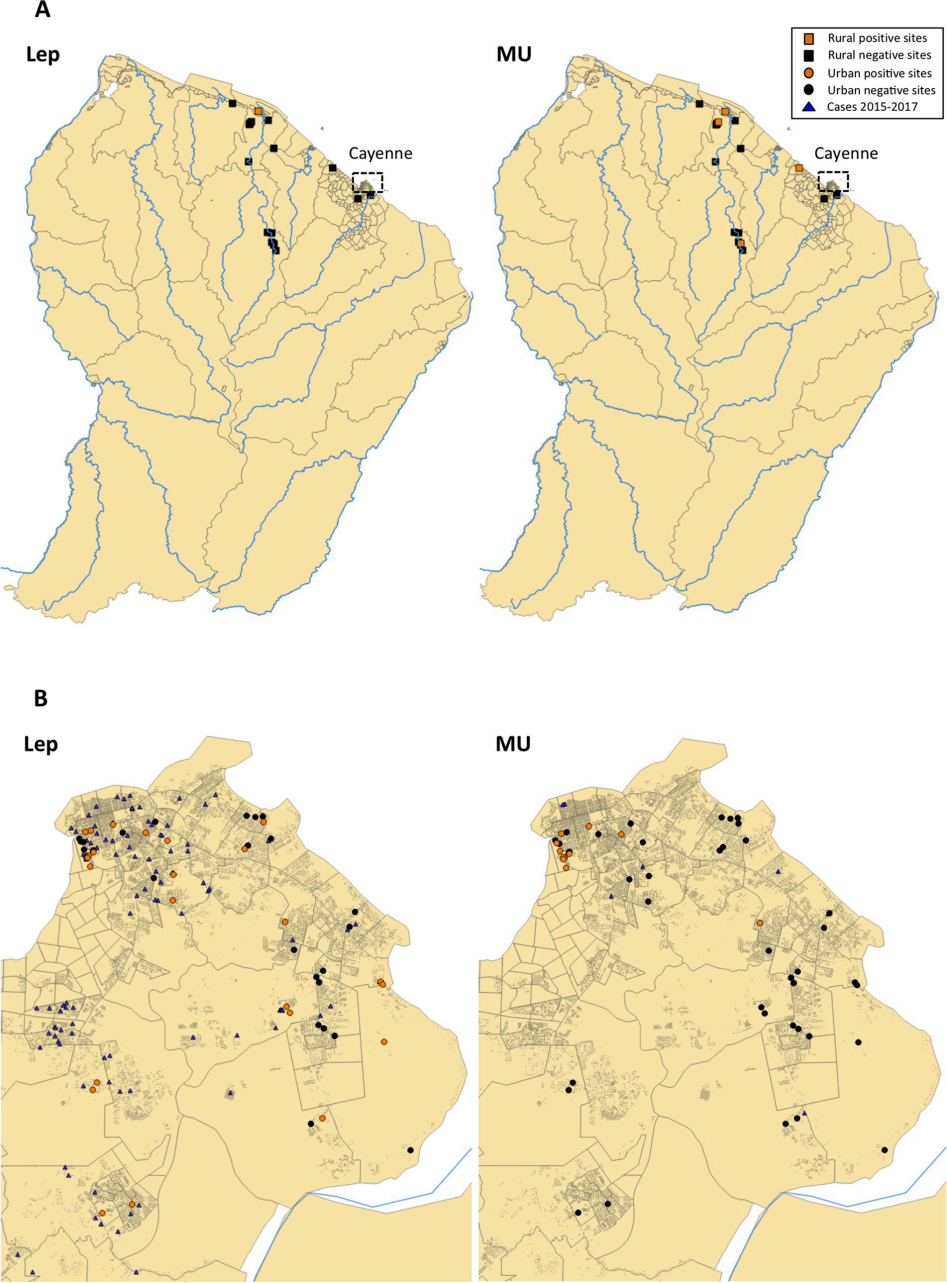
Localization of environmental sites tested for the presence of *Leptospira sp*. and *M*. *ulcerans* DNA. A: Rural sites sampled from november 2015 to march 2017; B: Urban sites localized in Cayenne and sampled from september 2016 to october 2017. All sites were surveyed during the dry and the rainy seasons. Lep: *Leptospira sp*. DNA; MU: *M*. *ulcerans* DNA. A site was considered positive when the bacteria’s DNA was found at least at one time period. For *Leptospira sp*. quantitative data were considered positive for CT-values < 40, whilst for *M*. *ulcerans* CT-threshold was set at 38. Maps were created with QGIS (version 2.18.20).

Among the 18 rural sites tested for the presence of *Leptospira sp*. DNA, only one site (R9) was found positive for this pathogen (Fig 2A, Table 1). Moreover, this site was found positive at only one sampling period, in february 2017, leading to a total of 1/201 (0.5%) sample recorded positive for *Leptospira sp*. DNA in rural areas in French Guiana. These 18 rural sites were also tested for the presence of *M*. *ulcerans* DNA and we found 4/18 sites (R5, R9, R13, R17) that harboured DNA of this mycobacteria, leading to a total of 12/201 (6%) samples that were positive for *M*. *ulcerans* DNA (Fig 2A, Table 1); site R5 was positive in november 2015 only, site R9 was positive in january, february, april, july, october and november 2016, site R13 was positive in december 2015 only, and site R17 in august, october and november 2016 (Supporting Information S1 Table). Therefore these results show a greater number of sites found positive for *M*. *ulcerans* in natural aquatic systems compared with *Leptospira sp.*. Also, *M*. *ulcerans* DNA was found in remote prestine sites with no human contact on the upper part of the Sinnamary River (R5), and one site (R9) was suitable for both pathogens.

In parallel, 51 urban water bodies were also sampled for water and sediments from september 2016 to october 2017 around Cayenne, Rémire-Montjoly and Matoury (Table 1). We found a total of 34/169 (20%) samples positive for *Leptospira sp*. DNA and 27/169 (16%) samples positive for *M*. *ulcerans* DNA (Fig 2B, Table 2). These results suggest that urban habitats are more favourable to the establishment of *Leptospira sp*. and *M*. *ulcerans*, which exhibited higher number of positive sites through time when compared with rural environments (20% *vs*. 0.5% and 16% *vs*. 6%, respectively).

### Effect of seasonality on the dynamics of both pathogens in the environment

The only rural site found positive for *Leptospira sp*. DNA corresponds to the rainy season (average rainfall of 270.4 mm, Table 2). In contrast, the majority of rural sites found positive for *M*. *ulcerans* DNA were positive during the dry periods (see Table 2). A total of 7/11 rural sites (64%) were found positive for *M*. *ulcerans* DNA during the dry season.

**Fig 3:**
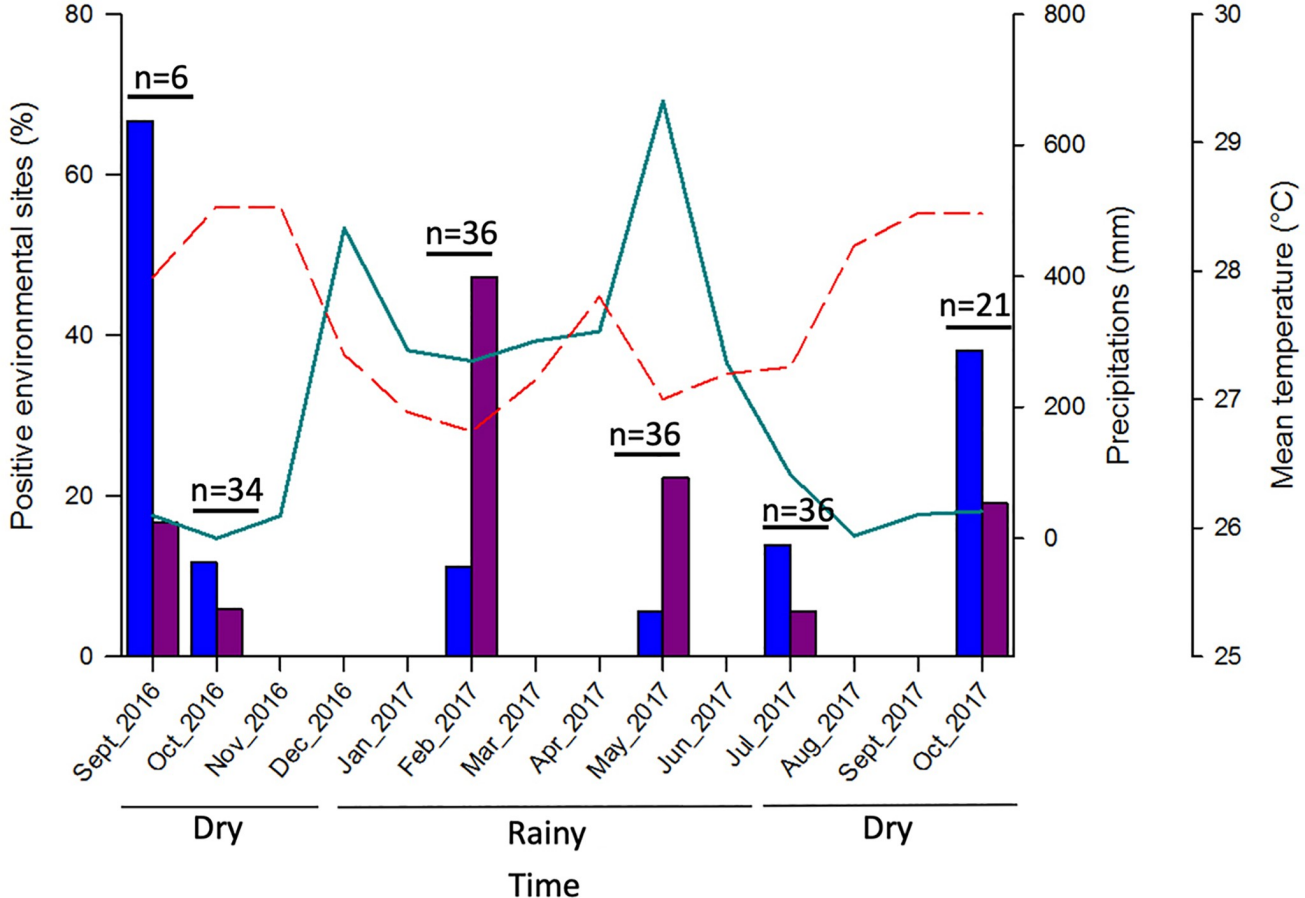
Prevalence of *Leptospira sp*. and *M*. *ulcerans* DNA in the environment through time. The prevalence of each bacterium in the environment was calculated based on the ratio between the number of positive urban sites and the total number of sites tested (%). Urban sites were sampled during the dry and the rainy season, allowing to follow the dynamics of each pathogen in space and time. Blue histograms show *M*. *ulcerans* positive sites, purple histograms represent *Leptospira sp*. positive sites, the green line represents the total precipitations per month (mm) and the red dashed line the mean temperature per month (°C). Precipitations and temperatures were recorded by Météo France in Cayenne (http://www.meteofrance.com/climat/outremer/cayenne).

**Fig 4:**
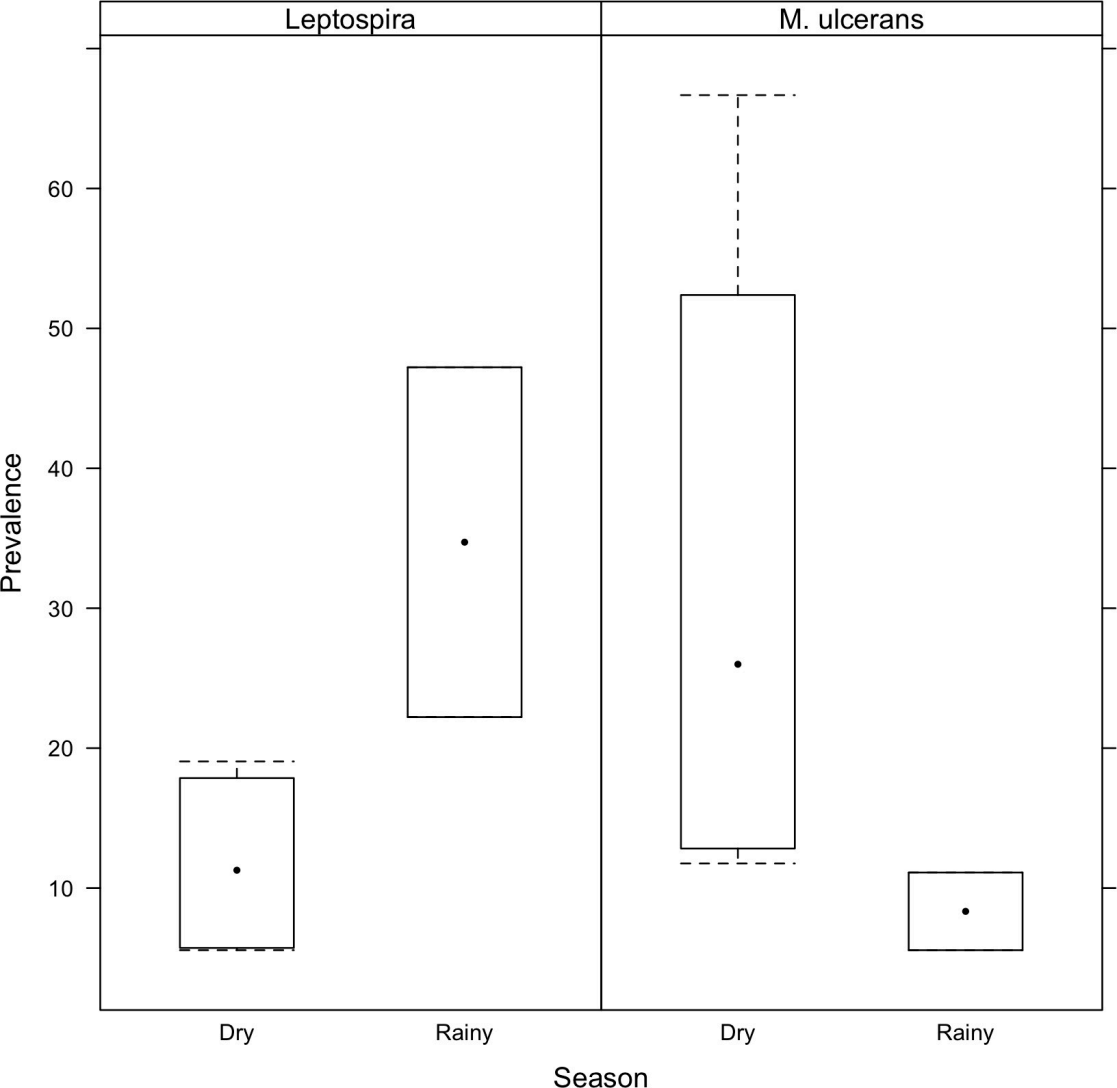
Prevalence of *Leptospira sp*. and *M*. *ulcerans* DNA in environmental sites during the dry and rainy seasons. The prevalence of each bacterium in the environment was calculated based on the ratio between the number of positive urban sites and the total number of sites tested (%). Using raw data we performed a logistic binomial (positive/negative) regression (R version 3.5.1) that clearly showed significant differences between the dry and the rainy seasons for both pathogens, whith *Leptospira sp*. being more prevalent (5.20 times higher; p-value = 0.00012) in the environment during the rainy season and *M*. *ulcerans* during dry periods (2.86 times higher; p-value = 0.0339).

Among the urban sites, the prevalence of *Leptospira sp*. DNA increased during the rainy season to 47.2% and 22.2% in February and May respectively, and reached its minimum during the dry period (Supporting Information S1 Map, Table 2). Although *M*. *ulcerans* DNA prevalence was more stable in the environment across seasons, most positive sites were observed during the dry period from 11.8% to 66.7% depending on the months (Supporting Information S2 Map, Table 2). Our results showed a correlation between the prevalences of each pathogen in the environment and seasonality, such that the prevalence of pathogenic leptospires was enhanced by rainfall while *M*. *ulcerans*’s prevalence in aquatic sites was more related to drought (Fig 3). These observations were confirmed by the binomial regression models that showed that the environmental dynamics of *Leptospira sp*. and *M*. *ulcerans* were significantly different between the dry and the rainy seasons. Indeed, environmental sites had 2.86 times (95% CI 1.14–8.19; p-value = 0.0339) higher odds of *M*. *ulcerans* positivity during the dry season, while *Leptospira sp*. had 5.20 times (95% CI 2.32–12.63; p-value = 0.00012) higher odds of being detected in the rainy season (Fig 4).

## Discussion

### Impact of urbanization on disease risk

Here our aim was to follow and describe the environmental dynamics of two aquatic bacteria, potentially pathogenic to animals and/or humans, across the French Guiana territory including the Amazon tropical rainforest. Our environmental survey describe the presence of both pathogens in rural and urban sites, with *M*. *ulcerans* being more often encountered in natural (undisturbed) rural habitats compared with *Leptospira sp.*. As proposed before, our results suggest the ubiquitous nature of the mycobacterium *M*. *ulcerans* in the environment [19]. According to Combe et al. [8], this pathogen is likely to be widely distributed in suitable natural aquatic systems and, under specific environmental conditions could become more abundant in the system, resulting an increased risk of Buruli ulcer emergence. Whilst *M*. *ulcerans* DNA was more often found in rural aquatic sites than *Leptospira sp*., we observed that one site (R9) was suitable for both pathogens, which was the only rural site located close to poor human settlements and frequently visited by livestock, such as cows and pigs, and domestic animals such as dogs, cats and poultry. This suggest that the presence of *Leptospira sp*. in rural environments would need the presence and activity of humans, livestock and/or domestic animals, that would represent sustainable host populations for this pathogen. The presence of *Leptospira sp*. in peri-domestic water samples from rural households has already been reported in southern Chile, with the presence of dogs and a high density of rodent populations being associated with positive puddles in the lower income households [26]. Pathogenic leptospires could be permanently present in the aquatic environment, and more specifically in watered soils as recently showed in New Caledonia [12], but only detected by qPCR when their abundance is increased by the proximity with human settlements and the availability of animal hosts that could also represent more attractive areas for rodent populations harbouring and sharing the bacteria in surrounding aquatic sites [5].

Also, the results suggest that modified urban environments are more favourable to the establishment of *Leptospira sp*. and *M*. *ulcerans*, with higher positivity for both pathogens when compared with rural environments (20% *vs*. 0.5% and 16% *vs*. 6%, respectively). Here the difference in the number of sampling sites and the different sampling scale in rural (18 water bodies, 17 months, 1 samples, n = 306) *vs* urban areas (51 water bodies, 6 months, 1 sample, n = 306), could constrain our interpretation when comparing the presence of both pathogens in these environments. However, in French Guiana most of the population live along the coastline and only 5 centers can be considered as urban (based on infrastructure development, population density, etc.): Cayenne (2441,27 inhabitants/km^2^), Rémire-Montjoly (519,97 inhabitants/km^2^), Matoury (236,37 inhabitants/km^2^), Kourou (12,14 inhabitants/km^2^) and Saint-Laurent du Maroni (9,03 inhabitants/km^2^) [27]. Our sampling effort covered 3 of these urban centers (Cayenne, Rémire-Montjoly and Matoury), and each show higher number of positive sites for *Leptospira sp*. and *M*. *ulcerans* when compared to rural sites. Moreover our sampling stategy was designed to reflect the differences in number of human cases in urban *vs* rural settings, with 99,3% and 8% of human cases for leptospirosis and Buruli ulcer occuring in urban populations versus 0,73% and 27,3% of cases occuring in rural ones, respectively. As the number of human cases was extremely low in rural settings it was important to have more regular sampling and over a wider geographical area in order to not miss any environmental signal. In urban settings this was not an issue and as such we adopted seasonal based sampling over the whole urban habitat. Therefore our results are consistent with the distribution of human cases across French Guiana, with much less cases in rural than in urban areas [20]. Therefore, we do not observe in villages located in the countryside clusters of human cases as observed in urban environments. In addition, a lack of replicates (1 sample/site) could potentially be a limit as sometimes many samples at a site can result in a single positive sample, or even none (from our field experience). However, our results show that even with such sampling effort (1 sample/site), we had no difficulties in detecting the presence of both pathogens in the environment, and confirmed their presence across seasons similar to what was previously reported on multiple samples [6,9,14]. Therefore, the sampling effort did not overestimated the presence of these pathogens.

In urban environments these pathogens seems to establish, colonize and share similar ecological niches (e.g. benthic algae and watered soils from 1–5 cm depth for pathogenic leptospire [12]; algae biofilm and watered soils from 1–10 cm depth for *M*. *ulcerans*, personnal data), habitats that also exist in rural settings but which are for some biotic reasons less favourable to their development. Casanovas-Massana and collaborators (2018) found that both sewage water and standing water were reservoirs for pathogenic *Leptospira sp*. in a urban area in Salvador, Brazil [5]. It shows that simplified urban ecosystems with less predators to rodents, low level trophic networks due to pollution and increased contact with humans would favour leptospirosis and Buruli ulcer emergence and transmission.

Other diseases are also known to be more prevalent in urban areas, such as dengue fever due to the availability of suitable human-created micro-environments for *Aedes sp*. mosquitoes breeding and eggs laying [28], or even for water-borne and enteric diseases with a oral-fecal transmission in areas with poor sanitation infrastructure [7]. Since most human emerging and/or re-emerging infectious diseases are zoonotic, increased urbanization in developing tropical countries would tend to increase the frequency of contact between wildlife and humans, representing an increased risk of disease emergence [7]. Indeed, drivers such as land-use changes (i.e. from natural toward deforested areas, agriculture intensification, road building) modify the ecology of the pathogen-vector-host, as well as increase contact between pathogen-vector and humans (i.e. increase human population densities, pollution, unsanitary conditions). The prevention of aquatic bacterial diseases emergence and transmission in tropical areas, where millions of people are currently living and where half of the world’s population will live by 2050 [7], would necessarily result from improved infrastructure and sanitation in impoverished urban areas of developing countries.

### Effect of seasonality on the dynamics of both pathogens in the environment

Here we showed a synchronisation between the presence of each pathogen in the environment and seasonality, with higher number of positive sites for pathogenic leptospires during the rainy season while *M*. *ulcerans*’s presence in aquatic sites was more related to drought (Fig 3, Fig 4). Previous findings indicated that leptospirosis outbreaks frequently occurred during periods of seasonal rainfall and flooding events in endemic areas [5,15,16,17]. Moreover, seasonal (i.e. rain *vs* drought) conditions leading to increased human exposure to contaminated water are known to be important drivers for leptospirosis transmission, and the proximity of households to open drainage systems and direct contact with sewage, flooding water and runoff were associated with increased risk of infection [29–33]. Similarly, a recent study conducted in Brazil found higher bacterial concentrations in urban environmental sites (sewage and standing water) during the rainy season when compared with the dry period, indicating thus a seasonal effect [5]. Also, the link between Buruli ulcer cases and seasonal patterns has been identified in several studies, showing that Buruli cases occurred during the dry season that followed rainfall events [reviewed in reference 8]. These observations were confirmed by long-term time series of Buruli ulcer cases [9] and climatic models [34,35] that revealed robust correlations between disease incidence and seasonality, with the disease being reported in French Guiana after dry periods following periods of heavy rainfall. Moreover in French Guiana Morris et al. (2014) have linked Buruli ulcer cases with extreme weather events such as La Niña, that are responsible to cause short dry periods during the rainy season [9].

Here the most interesting findings rely on the comparison of the environmental dynamics of the two aquatic bacterial pathogens in the same unit of time and space across both rural and urban environments. Indeed, we found that both pathogens are ubiquitously distributed in aquatic sites (persistent with low burden) although this seems more obvious for *M*. *ulcerans* in rural settings, and are both able to survive under similar ecological conditions. However, our seasonal survey clearly showed that the presence of each pathogen in the environment were heterogeneous and depended on different climatic patterns; whilst the presence of pathogenic leptospires in the environment was enhanced by rainfall, *M*. *ulcerans* emergence was boosted by drought that followed rainfall and flooding events. These results suggested that in French Guiana the infectious risk for each disease does not occur at the same period of the year, and is constrained by seasonality. Such local prevalence variability due to seasonal patterns might result from the pathogen’s life cycle or the dynamics of reservoir and/or host populations.

Looking at the spatial distribution of *Leptospira sp*. and *M*. *ulcerans* in the urban environment show that positive sites for *Leptospira sp*. are much more widely distributed when compared with *M*. *ulcerans* positive sites that are locally constrained within small neighborhoods (Fig 2B). We propose that such difference in the spatial distribution of both bacteria across urban sites could also be explained by the mode of dissemination of each pathogen in the environment (i.e. environmental dissemination of *M*. ulcerans *vs*. vector-borne/animal-borne for *Leptospira sp*.). During rainfall with increased oxygen levels and alkaline *pH* (up to *pH* 8.0), low salt concentrations, and/or the dilution of sewage toxic compounds, *Leptospira sp*. flourish [5,12,36] and are ingested by rodents and other carrier mammals. Flooded urban habitats, favour the re-distribution of rodents across cities, thus dispersing leptospires from one site to another mainly *via* urinary excretions. It does result in an increase of cases during the rainy season over a wide urban range. Such patterns have been also observed in other settings around the world where leptospirosis epidemics occurred in the rainy season that followed heavy rainfall [37,38,39]. Alternatively, Ferreira de Albuquerque et al. (2017) reported that capybaras (*Hydrochoerus hydrochaeris*) were massively infected by leptospires in the western Amazon region [40]. These small mammals are present in urban areas in French Guiana and could thus further play a role in the environmental dissemination of pathogenic leptospires. Unfortunately studies on rodent infections with *Leptospira sp*. are very scarce and old in French Guiana [41]. In contrast, the ecological conditions favouring *M*. *ulcerans* emergence in the environment are known to rely on higher water temperature, low *pH*, low oxygen levels and the presence of algal biofilm, conditions typically encountered during the dry period in the tropics [reviewed in reference 8]. In addition, *M*. *ulcerans* is not transmitted by a specific vector, but rather was found to be associated with a large range of aquatic invertebrates. Several studies showed that aquatic organisms of low/mid trophic level usually exhibit greater bacterial loads compared with organisms of a higher trophic level [reviewed in reference 8]. For instance *M*. *ulcerans* seems to have a specific association with gathering collectors and filter feeders. After anthropogenic (i.e. deforestation) or natural (i.e. changes in weather, flooding) changes, stagnant water bodies are prone to rapid local abiotic changes (temperature, *pH*, oxygen, etc.) associated with a rapid turnover of the biotic community (i.e. changes in functional diversity) and leading to an increase in favourable hosts harbouring *M*. *ulcerans* [8]. Based on the current knowledge on *M*. *ulcerans* ecology it appears clearly that its distribution is locally constrained by habitat and aquatic hosts communities highlighting the relative clustering of human cases within urban units in French Guiana.

Whilst the wide presence of *M*. *ulcerans* along French Guiana’s coast was already known, this is the first survey that screened for the presence of pathogenic leptospires across the territory (including the Amazon tropical rainforest). Until recently it was assumed that there were few leptospirosis cases in French Guiana, compared to West Indies for instance, probably because of the acidity of the soil across the Guiana shield preventing bacterial development. However our results clearly showed that *(i)* both pathogens are present in the environment in French Guiana, and *(ii)* urbanization and seasonality are two important factors underlying Buruli ulcer and leptospirosis emergence. Also we propose that the mode of transmission (i.e. generalist *vs*. vector-borne) of environmental pathogens might have a detrimental role in disseminating the infectious agent in the environment. To better monitor diseases emergence, we suggest that future studies should focus on determining which specific socio-economic and environmental factors are underlying the spatio-temporal distribution of emerging infectious pathogens.

## Supporting information

S1 Table Quantitative-PCR (qPCR) values for each site through the environmental survey. For each site LipL32 (*Leptospira sp*. DNA detection), *IS*2404 and KR (*M*. *ulcerans* DNA identification) cycle-treshold (CT) values are indicated. For rural sites, only positive sites for *Leptospira sp*. and/or *M*. *ulcerans* DNA are indicated. Sites with no CT-values observed are indicated by a dash.
(DOCX)

S1 Map Spatio-temporal dynamics of pathogenic leptospires in urban aquatic sites in Cayenne. Sites were sampled at 6 time periods, corresponding to the dry and rainy seasons, and were tested for the presence of *Leptospira sp*. DNA by qPCR targetting LipL32 sequence of pathogenic strains. A site was considered positive for a CT-value < 40. A: september 2016, B: october 2016, C: february 2017, D: may 2017, E: july 2017, F: october 2017. Maps were created with QGIS (version 2.18.20).
(TIFF)

S2 Map Spatio-temporal dynamics of *M*. *ulcerans* DNA in urban aquatic sites in Cayenne. Sites were sampled at 6 time periods, corresponding to the dry and rainy seasons, and were tested for the presence of *M*. ulcerans DNA by qPCR targetting IS2404 and KR sequences. A site was considered positive when both markers had a CT-value < 38. A: september 2016, B: october 2016, C: february 2017, D: may 2017, E: july 2017, F: october 2017. Maps were created with QGIS (version 2.18.20).
(TIFF)
