# Supplementary material for: Comparison of Mycobacterium ulcerans (Buruli ulcer) and Leptospira sp. (Leptospirosis) dynamics in urban and rural settings
Source: PLoS Negl Trop Dis. 2019 Jan 7;13(1):e0007074. doi: 10.1371/journal.pntd.0007074 (PMC6336349; doi:10.1371/journal.pntd.0007074)
Supplement: S1 Table — For each site LipL32 (Leptospira sp. DNA detection), IS2404 and KR (M. ulcerans DNA identification) cycle-treshold (CT) values are indicated. For rural sites, only positive sites for Leptospira sp. and/or M. ulcerans DNA are indicated. Sites with no CT-values observed are indicated by a dash. (DOCX) [file pntd.0007074.s001.docx]

**Supporting Information S1 Table. Quantitative-PCR (qPCR) values for each site through the environmental survey.** For each site LipL32 (*Leptospira sp.* DNA detection), *IS*2404 and KR (*M. ulcerans* DNA identification) cycle-treshold (CT) values are indicated. For rural sites, only positive sites for *Leptospira sp.* and/or *M. ulcerans* DNA are indicated. Sites with no CT-values observed are indicated by a dash.

|  |  | ***Leptospira sp.*** | ***M. ulcerans*** | ***M. ulcerans*** | |
| --- | --- | --- | --- | --- | --- |
| **Site** | **Sampling period** | **LipL32 CT-value** | ***IS*2404 CT-value** | | **KR CT-value** |
| U1 | september 2016 | 37,8876 | 35,7074 | | 35,6104 |
| U2 | september 2016 | - | 38,9165 | | - |
| U3 | september 2016 | - | 37,9248 | | - |
| U4 | september 2016 | - | 35,7715 | | 36,4725 |
| U5 | september 2016 | - | 37,6014 | | - |
| U6 | september 2016 | - | 37,5669 | | 35,0692 |
| U1 | october 2016 | - | - | | - |
| U4 | october 2016 | - | 35,1571 | | 33,2899 |
| U6 | october 2016 | - | 35,8796 | | - |
| U7 | october 2016 | - | - | | - |
| U8 | october 2016 | - | 40,6411 | | - |
| U9 | october 2016 | - | - | | - |
| U10 | october 2016 | 42,447 | - | | - |
| U11 | october 2016 | - | - | | - |
| U12 | october 2016 | - | - | | - |
| U13 | october 2016 | - | - | | - |
| U14 | october 2016 | - | - | | - |
| U15 | october 2016 | - | - | | - |
| U16 | october 2016 | - | - | | - |
| U17 | october 2016 | - | - | | - |
| U18 | october 2016 | - | - | | - |
| U19 | october 2016 | - | - | | - |
| U20 | october 2016 | 35,9712 | - | | - |
| U21 | october 2016 | - | - | | - |
| U22 | october 2016 | - | - | | - |
| U23 | october 2016 | - | - | | - |
| U24 | october 2016 | - | - | | - |
| U25 | october 2016 | 38,7683 | - | | - |
| U26 | october 2016 | - | - | | - |
| U27 | october 2016 | - | 36,8359 | | - |
| U28 | october 2016 | - | - | | - |
| U29 | october 2016 | - | - | | - |
| U30 | october 2016 | 43,1007 | 37,7976 | | - |
| U31 | october 2016 | - | 31,1005 | | 31,8121 |
| U32 | october 2016 | - | - | | - |
| U33 | october 2016 | - | 37,761 | | 32,9419 |
| U34 | october 2016 | - | - | | - |
| U35 | october 2016 | - | 34,1594 | | 34,0048 |
| U36 | october 2016 | - | - | | - |
| U37 | october 2016 | - | - | | - |
| U1 | february 2017 | - | - | | - |
| U4 | february 2017 | 38,57 | 38,8652 | | 33,4917 |
| U5 | february 2017 | 38,8605 | 37,0531 | | 35,772 |
| U6 | february 2017 | - | 39,4452 | | 36,8646 |
| U8 | february 2017 | 36,6397 | 38,9801 | | - |
| U9 | february 2017 | 38,271 | - | | - |
| U10 | february 2017 | - | - | | - |
| U12 | february 2017 | - | - | | - |
| U13 | february 2017 | - | - | | - |
| U14 | february 2017 | - | - | | - |
| U16 | february 2017 | 36,4632 | - | | - |
| U17 | february 2017 | 35,3611 | - | | - |
| U22 | february 2017 | - | - | | - |
| U24 | february 2017 | 32,5809 | - | | - |
| U26 | february 2017 | - | - | | - |
| U28 | february 2017 | - | - | | - |
| U30 | february 2017 | - | - | | - |
| U31 | february 2017 | 44,2486 | 39,0171 | | 35,4029 |
| U32 | february 2017 | 43,5904 | 31,2398 | | 31,5257 |
| U33 | february 2017 | 39,4856 | 34,4634 | | 35,6542 |
| U34 | february 2017 | 36,1903 | - | | - |
| U35 | february 2017 | - | - | | - |
| U36 | february 2017 | - | 36,6064 | | 34,4791 |
| U38 | february 2017 | - | - | | - |
| U39 | february 2017 | - | - | | - |
| U40 | february 2017 | 33,9502 | - | | - |
| U41 | february 2017 | 41 | - | | - |
| U42 | february 2017 | - | - | | - |
| U43 | february 2017 | 37,4816 | - | | - |
| U44 | february 2017 | 34,5425 | - | | - |
| U45 | february 2017 | 36,5826 | - | | - |
| U46 | february 2017 | - | - | | - |
| U47 | february 2017 | 38,3824 | - | | - |
| U48 | february 2017 | 37,3923 | - | | - |
| U49 | february 2017 | 37,9041 | - | | - |
| U50 | february 2017 | 38,5795 | - | | - |
| U1 | may 2017 | 39,5049 | - | | - |
| U4 | may 2017 | 38,2969 | 36,2835 | | 35,8835 |
| U5 | may 2017 | 39,605 | - | | - |
| U6 | may 2017 | 40,5342 | - | | - |
| U8 | may 2017 | 37,4469 | - | | - |
| U9 | may 2017 | 40,5399 | - | | - |
| U10 | may 2017 | 38,3566 | - | | - |
| U12 | may 2017 | 42,3524 | - | | - |
| U13 | may 2017 | 44,3051 | - | | - |
| U14 | may 2017 | - | - | | - |
| U16 | may 2017 | - | - | | - |
| U17 | may 2017 | - | - | | - |
| U22 | may 2017 | - | - | | - |
| U24 | may 2017 | - | - | | - |
| U26 | may 2017 | 35,3875 | - | | - |
| U28 | may 2017 | 41,088 | - | | - |
| U30 | may 2017 | - | - | | - |
| U31 | may 2017 | - | - | | - |
| U32 | may 2017 | - | 38,2135 | | 36,9663 |
| U33 | may 2017 | - | - | | - |
| U34 | may 2017 | - | - | | - |
| U35 | may 2017 | 40,3398 | - | | - |
| U36 | may 2017 | 39,8582 | - | | - |
| U38 | may 2017 | 36,767 | - | | - |
| U39 | may 2017 | - | - | | - |
| U40 | may 2017 | - | - | | - |
| U41 | may 2017 | - | - | | - |
| U42 | may 2017 | - | - | | - |
| U43 | may 2017 | 43,624 | - | | - |
| U44 | may 2017 | 41,2216 | - | | - |
| U45 | may 2017 | 40,2953 | - | | - |
| U46 | may 2017 | - | - | | - |
| U47 | may 2017 | - | - | | - |
| U48 | may 2017 | - | - | | - |
| U49 | may 2017 | - | - | | - |
| U50 | may 2017 | - | - | | - |
| U1 | july 2017 | - | - | | - |
| U4 | july 2017 | - | 33,9693 | | 35,9505 |
| U5 | july 2017 | - | - | | - |
| U6 | july 2017 | - | 34,3667 | | 34,3705 |
| U8 | july 2017 | - | - | | - |
| U9 | july 2017 | - | - | | - |
| U10 | july 2017 | - | - | | - |
| U12 | july 2017 | 42,6149 | - | | - |
| U13 | july 2017 | - | - | | - |
| U14 | july 2017 | - | - | | - |
| U16 | july 2017 | - | 39,2742 | | 38,0961 |
| U17 | july 2017 | 33,818 | - | | - |
| U18 | july 2017 | - | - | | - |
| U22 | july 2017 | - | - | | - |
| U24 | july 2017 | - | - | | - |
| U26 | july 2017 | - | - | | - |
| U28 | july 2017 | - | - | | - |
| U30 | july 2017 | 40,13 | - | | - |
| U31 | july 2017 | - | 33,7896 | | 33,6138 |
| U32 | july 2017 | - | 33,3047 | | 34,1016 |
| U33 | july 2017 | - | 36,544 | | 37,2628 |
| U34 | july 2017 | - | 39,3107 | | - |
| U35 | july 2017 | - | 38,2754 | | - |
| U36 | july 2017 | - | - | | - |
| U38 | july 2017 | - | - | | - |
| U39 | july 2017 | - | - | | - |
| U40 | july 2017 | - | - | | - |
| U41 | july 2017 | - | - | | - |
| U42 | july 2017 | - | - | | - |
| U43 | july 2017 | - | - | | - |
| U44 | july 2017 | - | - | | - |
| U45 | july 2017 | - | - | | - |
| U47 | july 2017 | - | - | | - |
| U48 | july 2017 | 39,9473 | - | | - |
| U49 | july 2017 | - | - | | - |
| U50 | july 2017 | - | - | | - |
| U1 | october 2017 | - | - | | - |
| U4 | october 2017 | - | 31,6379 | | 35,4965 |
| U5 | october 2017 | - | 33,9943 | | 35,2196 |
| U6 | october 2017 | - | 29,6619 | | 34 |
| U8 | october 2017 | - | 28,6526 | | 32,4996 |
| U9 | october 2017 | - | - | | - |
| U10 | october 2017 | - | - | | - |
| U16 | october 2017 | - | - | | - |
| U17 | october 2017 | - | - | | - |
| U20 | october 2017 | 38,9319 | - | | - |
| U22 | october 2017 | - | 38,3833 | | - |
| U30 | october 2017 | 36,3807 | 37,5246 | | 36,8345 |
| U31 | october 2017 | - | - | | - |
| U32 | october 2017 | - | 32,3724 | | 35,4099 |
| U33 | october 2017 | - | 34,5383 | | 39,2105 |
| U34 | october 2017 | - | - | | - |
| U35 | october 2017 | - | 31,5226 | | 37,0277 |
| U36 | october 2017 | - | 41,6297 | | - |
| U43 | october 2017 | 39,7408 | - | | - |
| U45 | october 2017 | 38,4815 | - | | - |
| R5 | november 2015 | - | 36,958 | | 35,94635 |
| R2 | november 2015 | - | 39,11395 | | 33,9508 |
| R13 | december 2015 | - | 38,2902 | | 34,8201 |
| R16 | december 2015 | - | 39,0895 | | - |
| R9 | january 2016 | - | 34,3172 | | 34,48715 |
| R17 | january 2016 | - | 39,3209 | | - |
| R9 | february 2016 | - | 35,29086667 | | 35,29625 |
| R1 | march 2016 | - | 30,7059 | | - |
| R9 | march 2016 | - | 37,2868 | | - |
| R9 | march 2016 | - | 38,9385 | | - |
| R9 | april 2016 | - | 34,5676 | | 37,4611 |
| R9 | april 2016 | - | 37,27755 | | - |
| R9 | april 2016 | - | 38,0002 | | - |
| R9 | may 2016 | - | 39,8963 | | - |
| R9 | june 2016 | - | 39,7509 | | - |
| R9 | june 2016 | - | 37,9304 | | - |
| R9 | july 2016 | - | 35,3552 | | 34,4439 |
| R9 | august 2016 | - | 37,7176 | | - |
| R9 | august 2016 | - | 38,647 | | - |
| R9 | august 2016 | - | 38,1887 | | - |
| R17 | august 2016 | - | 36,1011 | | 35,0402 |
| R9 | september 2016 | - | 36,9261 | | - |
| R17 | september 2016 | - | 38,2981 | | - |
| R17 | september 2016 | - | 38,8125 | | - |
| R1 | october 2016 | - | 38,436 | | - |
| R9 | october 2016 | - | 35,3471 | | 34,1823 |
| R17 | october 2016 | - | 37,2776 | | 34,3372 |
| R1 | november 2016 | - | 38,1358 | | - |
| R9 | november 2016 | - | 34,0368 | | 34,2948 |
| R9 | november 2016 | - | 37,712 | | 35,9882 |
| R17 | november 2016 | - | 37,2832 | | - |
| R17 | november 2016 | - | 39,9913 | | 35,652 |
| R17 | november 2016 | - | 32,9656 | | 32,7731 |
| R9 | february 2017 | 39,2551 | - | | - |
| R17 | february 2017 | - | 40,2466 | | - |
