# Supplementary figures and images for: Comparison of Mycobacterium ulcerans (Buruli ulcer) and Leptospira sp. (Leptospirosis) dynamics in urban and rural settings
Source: PLoS Negl Trop Dis. 2019 Jan 7;13(1):e0007074. doi: 10.1371/journal.pntd.0007074 (PMC6336349; doi:10.1371/journal.pntd.0007074)

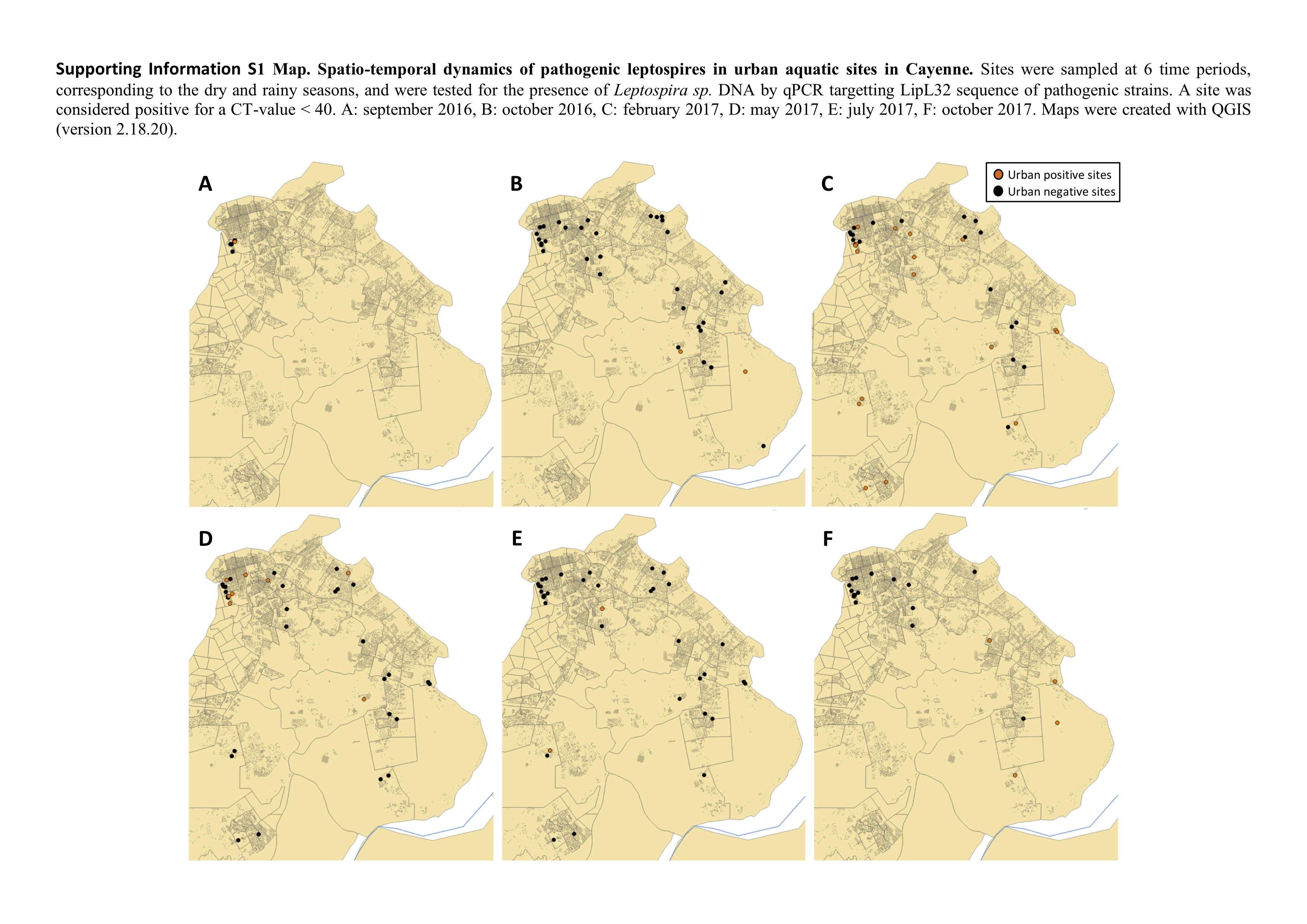

Supplement: S1 Map — Sites were sampled at 6 time periods, corresponding to the dry and rainy seasons, and were tested for the presence of Leptospira sp. DNA by qPCR targetting LipL32 sequence of pathogenic strains. A site was considered positive for a CT-value < 40. A: september 2016, B: october 2016, C: february 2017, D: may 2017, E: july 2017, F: october 2017. Maps were created with QGIS (version 2.18.20). (TIFF) [file pntd.0007074.s002.TIFF]

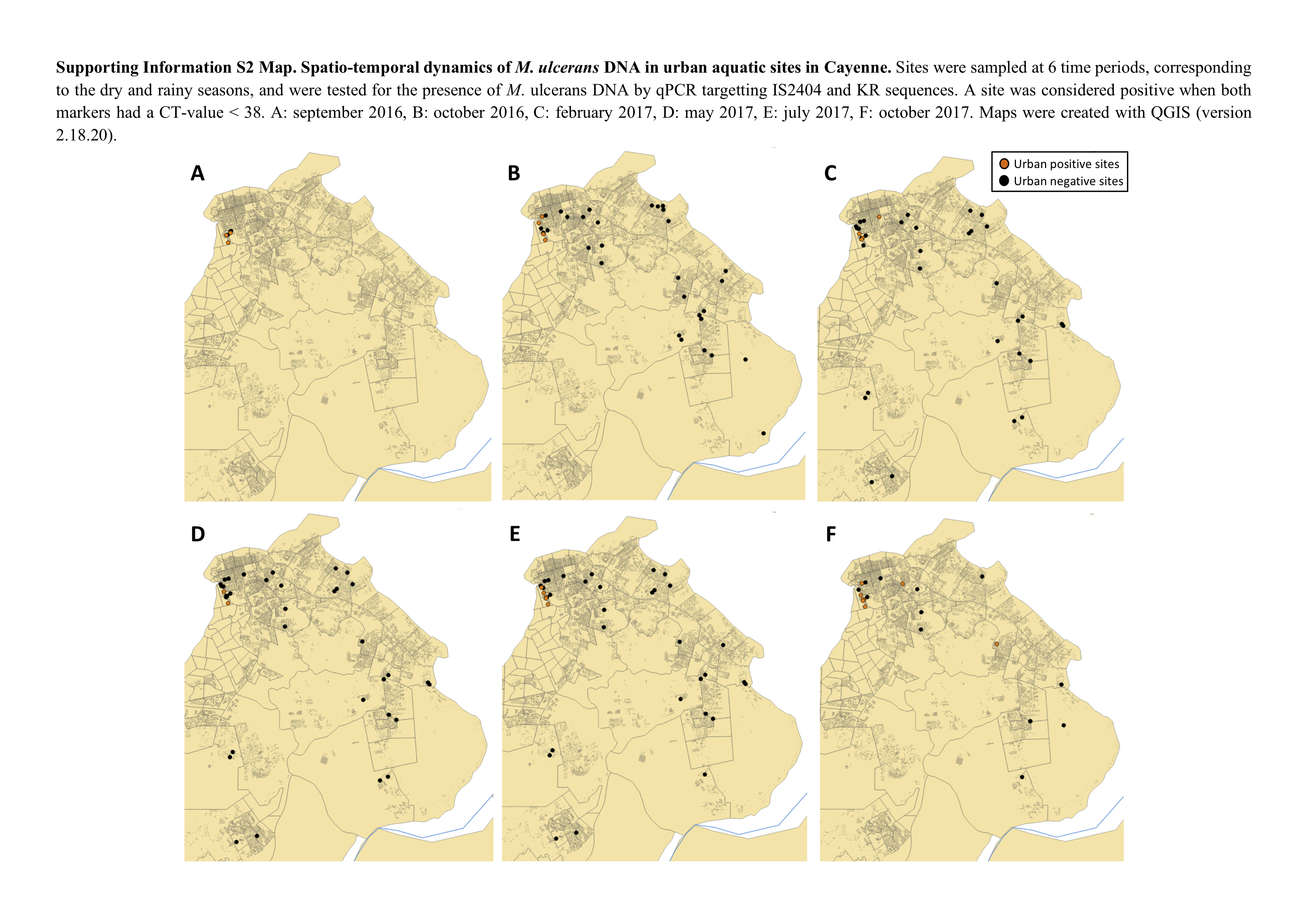

Supplement: S2 Map — Sites were sampled at 6 time periods, corresponding to the dry and rainy seasons, and were tested for the presence of M. ulcerans DNA by qPCR targetting IS2404 and KR sequences. A site was considered positive when both markers had a CT-value < 38. A: september 2016, B: october 2016, C: february 2017, D: may 2017, E: july 2017, F: october 2017. Maps were created with QGIS (version 2.18.20). (TIFF) [file pntd.0007074.s003.TIFF]
